# Níveis de Tiol Sérico e Homeostase Tiol/Dissulfeto em Pacientes com Doença Valvar Mitral Reumatismal e em Sujeitos Saudáveis

**DOI:** 10.36660/abc.20200161

**Published:** 2021-07-27

**Authors:** Ahmet Korkmaz, Birsen Doğanay, Funda Basyigit, Cem Çöteli, Abdulkadir Yildiz, Tugba Gursoy, Umit Guray, Ozgul Ucar Elalmis

**Affiliations:** 1 Ministry of Health Ankara City Hospital Department of Cardiology Ankara Turquia Ministry of Health Ankara City Hospital - Department of Cardiology, Ankara - Turquia; 2 Ozel Anadolu Hastanesi Kastamonu Turquia Ozel Anadolu Hastanesi - Cardiology Clinic,Kastamonu - Turquia

**Keywords:** Doenças Reumáticas, Estenose da Valva Mitral, Homeostase, Tiol/Dissulfeto, Ecocardiografia/métodos, Estresse Oxidativo

## Abstract

**Fundamento:**

A doença valvar mitral reumatismal (DVMR) é a apresentação mais comum das doenças cardíacas reumáticas (DCR). Os processos de inflamação e fibrose também têm papéis significativos em sua patogênese. Estudos recentes demonstram que os tióis e o tiol-dissulfeto são marcadores de stress oxidativo inéditos e promissores.

**Objetivos:**

O objetivo deste estudo foi avaliar diferenças entre os níveis de tiol sérico e de tiol-dissulfeto em pacientes com DVMR e no grupo de controle.

**Métodos:**

Noventa e dois pacientes com DVMR foram cadastrados no estudo. Cinquenta e quatro sujeitos saudáveis, e com correspondência de sexo e idade em relação ao grupo de estudo, também foram incluídos no estudo como um grupo de controle. Foram investigados os níveis de tiol nos pacientes com DVMR e o grupo de controle. Os p-valores menores que 0,05 foram considerados estatisticamente significativos.

**Resultados:**

Os pacientes com DVMR apresentaram pressão sistólica da artéria pulmonar (PSAP) e níveis de diâmetro do átrio esquerdo (AE) mais altos que os do grupo de controle. Os níveis de tiol nativo (407±83 μmol/L vs. 297±65 μmol/L, p<0,001) e tiol total (442±82 μmol/L vs. 329±65 μmol/L, p<0,001) são mais altos no grupo de controle. Níveis de dissulfeto (16,7±4,9 μmol/L vs. 14,8±3,7 μmol/L, p=0,011) são mais altos no grupo de pacientes com DVMR. Foi identificada uma correlação positiva entre as razões dissulfeto/tiol nativo e dissulfeto/tiol total com PSAP, diâmetro de AE, e gravidade da EMi. A razão dissulfeto/tiol total é significativamente mais alta em pacientes com EMi grave que em pacientes com EMi leve a moderada.

**Conclusões:**

Até onde se sabe, este é o único estudo que avaliou a homeostase tiol/dissulfeto como um preditor inédito, que está relacionado de forma mais próxima à DVMR e à gravidade da EMi.

## Introdução

A doença cardíaca reumática (DCR) é a doença cardiovascular mais amplamente observada em crianças e adolescentes.^1^ A doença valvar mitral reumatismal (DVMR) é a apresentação mais comum das DCR.^2^ O estudo em 2015 relatou que o número estimado de pacientes com DCR era de 33,4 milhões globalmente.^3^ No estudo PROVAR, os autores fizeram a triagem de 5996 alunos com mediana de idade de 11,9 (faixa de 9,0 a 15,0), com ecocardiograma. Os autores relataram que a prevalência de DCR era de 42/1000 em crianças brasileiras.^4^

A doença cardíaca reumática ocorre após uma reação autoimune, que é desencadeada por uma infecção estreptocócica do trato respiratório superior não tratada. Esse processo causa lesão valvar grave em sujeitos geneticamente suscetíveis.^5^ Apesar de a fisiopatologia das DCR ser desconhecida, há hipóteses de que várias reações inflamatórias e autoimunes, stress oxidativo, genes do sistema imune, e polimorfismos estejam relacionados às DCR.^5 - 9^

Os tióis são agentes antioxidantes cruciais na fisiologia humana. As concentrações de tiol são mais baixas em plasma. Isso acontece porque os tióis são compostos majoritariamente por albumina de plasma humano com tióis de baixo peso molecular, incluindo a cisteína (Cys), a homocisteína, a glutationa, a cisteinil glicina, e a γ-glutamilcisteína.^10^ Os processos oxidativos podem transformar os tióis em várias moléculas diferentes. O tiol-dissulfeto é um dos produtos das reações oxidativas em que os tióis estão envolvidos.^11^ A oxidação dos resíduos de Cys podem gerar a produção reversível de vários dissulfetos, tais como tióis de massa molecular baixa e moléculas de tiol proteico. Além disso, os resíduos de dissulfeto poderiam ser convertidos em grupos de tiol para manter a homeostase tiol/dissulfeto estável.^12^ Portanto, os tióis representam uma parte crucial da quantidade total de antioxidantes e têm um papel significativo no mecanismo antioxidante para espécies reativas oxidativas (ERO).^13^

Os estudos atuais demonstraram que a razão tiol-dissulfeto tinha um valor significativo como marcador de stress oxidativo promissor.^14 , 15^ Embora a importância do metabolismo de tióis tenha sido avaliada para procedimentos e doenças cardiovasculares diferentes, tais como o infarto do miocárdio ou cirurgia para ponte arterial, sua expressão em DCR não é conhecida.^14 , 16^ Neste estudo, devido ao componente fisiopatológico da DVR, buscamos avaliar os níveis de tiol em pacientes com DVMR e em sujeitos saudáveis.

## Métodos

Noventa e dois pacientes com DVMR que foram atendidos em nossa clínica entre abril de 2018 e dezembro de 2019 foram cadastrados no estudo. Cinquenta e quatro sujeitos saudáveis, e com correspondência em relação ao grupo de estudo, também foram incluídos no estudo como um grupo de controle. Foram considerados sexo, idade, índice de massa corporal (IMC), comorbidades, fração de ejeção do ventrículo esquerdo, e tabagismo para o pareamento dos grupos.

O termo de consentimento informado escrito foi obtido de todos os participantes. Os critérios de exclusão foram pacientes com síndrome de Marfan, válvula aórtica bicúspide, patologias de válvula mitral não reumáticas, ou cirurgia aberta prévia. Além disso, pacientes com doenças hepáticas, tireoidianas e renais, doenças hematológicas, doenças do tecido conjuntivo ou inflamatórias, qualquer histórico de câncer, e infecção aguda ou crônica também foram excluídos do estudo.

Os dados relevantes demográficos, antropométricos e de histórico médico foram registrados. Informações clínicas, tais como os fatores de risco coronarianos de Framingham (hipertensão (HT), diabetes mellitus (DM), tabagismo, hiperlipidemia, e histórico familiar de doença coronariana), também foram coletadas.

O ecocardiograma transtorácico bidimensional com Doppler colorido foi utilizado em todos os pacientes, utilizando-se ultrassom com transdutores de 2,5-MHz (Toshiba SSH160A). Foi utilizado o ecocardiograma modo M para medição do diâmetro atrial esquerdo, e os métodos de planimetria e meia-pressão foram utilizados para acessar a área da válvula mitral. O gradiente transmitral foi definido com um Doppler de onda contínua em um corte apical de quatro câmaras. O Doppler colorido foi realizado para detectar a presença e a gravidade da regurgitação mitral (RM). A pressão sistólica da artéria pulmonar foi medida por estudos de Doppler de onda contínua, utilizando-se a equação de Bernoulli. Os critérios para diagnóstico de DVMR incluem área da válvula mitral ≤ 2,5 cm^2^, a presença de espessamento de folhetos, fusão comissural, e alteração na área subvalvar, detectada por ecocardiograma.^17^

Foram coletadas amostras sanguíneas da veia antecubital durante a admissão no hospital. As amostras sanguíneas dos grupos de pacientes e controle foram coletadas pela manhã, após um período de jejum de 12 horas. Todas as amostras de sangue foram coletadas em tubos que não continham aditivos. Foi obtido soro após a centrifugação a 1500 g por 10 minutos, que foi armazenado a -80°C até a análise.

A homeostase tiol/dissulfeto foi realizada pelo procedimento determinado por Erel et al.,^18^ Em seguida, grupos de tiol funcional livre foram obtidos com a redução de ligações de dissulfeto. O boro-hidreto de sódio foi aplicado como redutor, e o redutor não utilizado foi retirado por formaldeído. Depois da reação com o ácido 5,5’-ditio-bis-(2-nitrobenzóico), todos os grupos de tiol, tanto os nativos quanto os reduzidos, foram definidos. A quantidade de dissulfeto dinâmico (−S-S) foi confirmada por metade da diferença entre tióis nativos e totais. Depois de avaliar a magnitude do tiol nativo (−SH) e do dissulfeto (−S-S), a razão dissulfeto/tiol nativo (−S-S−/−SH) foi calculada.^18^

### Análise estatística

A normalidade dos dados foi analisada utilizando-se o teste Kolmogorov-Smirnov. As variáveis quantitativas com distribuição normal foram apresentadas como média e desvio padrão. As variáveis com distribuição não normal foram apresentadas como mediana (faixa interquartil), e dados categóricos, como número e porcentagem. O teste t com amostras independentes foi utilizado para comparar os grupos em relação aos dados contínuos com distribuição normal, enquanto o teste U de Mann-Whitney foi realizado para variáveis com distribuição não normal. Os dados categóricos foram analisados usando o teste Qui-quadrado ou teste exato de Fisher. As relações entre as variáveis categóricas e numéricas foram analisadas pela análise da correlação de Spearman. As diferenças foram consideradas significativas no nível de p <0,05 de dois lados. Todas as análises estatísticas foram realizadas utilizando-se o programa Statistical Package for Social Sciences (SPSS) para Windows versão 22 (IBM SPSS Inc., Chicago, IL).

## Resultados

Um total de 146 sujeitos, dos quais 92 apresentavam DVMR, e 54 não, foi incluído no estudo. Os dados demográficos, clínicos e laboratoriais dos grupos estudados são apresentados na Tabela 1 . No grupo de pacientes com DVMR, um total de 22 (24%) eram do sexo masculino e 70 (76%) eram do sexo feminino, enquanto 15 (28%) pacientes eram do sexo masculino e 39 (72%) eram do sexo feminino no grupo de controle. A idade média era de 48±10 anos e 46,7±11,2 anos nos grupos de pacientes com DVMR e de controle, respectivamente. Não foram encontradas diferenças entre os grupos em termos de idade, sexo, IMC, HT, DM, tabagismo e outros parâmetros laboratoriais. Como esperado, os pacientes com DVMR apresentaram pressão sistólica da artéria pulmonar (PSAP) e níveis de diâmetro do átrio esquerdo (AE) mais altos que os do grupo de controle.

**Tabela 1:** – Características clínicas, demográficas de laboratoriais na linha de base dos pacientes com doença valvar mitral reumatismal

			Controle (n:54)	DVMR (n:92)	p
Idade (Mediana, FIQ1- FIQ3)			45 (39,3 - 54,5)	48 (42 - 55,8)	0,304 ^m^
Sexo (n,%)	Masculino		15 (28%)	22 (24%)	0,604 ^X^2^^
	Feminino		39 (72%)	70 (76%)	
DM (n,%)	Não		48 (89%)	88 (96%)	0,133 ^X^2^^
	Sim		6 (11%)	4 (4%)	
HT (n,%)	Não		46 (85%)	79 (86%)	0,492 ^X^2^^
	Sim		8 (15%)	13 (14%)	
Tabagismo, n(%)	Não		40 (74%)	58 (63%)	0,134 ^X^2^^
	Sim		14 (26%)	34 (37%)	
IMC (Mediana, FIQ1- FIQ3)			24 (22 - 25,8)	24 (21,9 - 25)	0,139 ^m^
Glicemia (mg/dL) (Mediana, FIQ1- FIQ3)			94 (88,5 - 102)	90 (21,9 - 25)	0,054 ^m^
Creatinina sérica (mg/dL) (Mediana, FIQ1- FIQ3)			0,8 (0,6 - 0,8)	0,7 (0,6 - 0,8)	0,128 ^m^
Hemoglobina (g/dL) (Mediana, FIQ1- FIQ3)			14 (13 - 14)	13,8 (12 - 14)	0,509 ^m^
Contagem WBC (x1000/mm3) (Mediana, FIQ1- FIQ3)			6,4 (5,9 - 7,3)	6,2 (6 - 7)	0,423 ^m^
Contagem de plaquetas (x1000/mm3) (Mediana, FIQ1- FIQ3)			300 (254,5 - 348,8)	301 (277 - 313)	0,766 ^m^
Colesterol total (mg/dL) (Mediana, FIQ1- FIQ3)			215 (195,3 - 235)	197 (195 - 225)	0,075 ^m^
LDL, (mg/dL) (Mediana, FIQ1- FIQ3)			120 (103,3 - 125)	114 (100 - 121)	0,086 ^m^
HDL, (mg/dL) (Mediana, FIQ1- FIQ3)			49 (45 - 55,8)	51 (45,8 - 72)	0,113 ^m^
Triglicérides (mg/dL) (Mediana, FIQ1- FIQ3)			150 (90,8 - 185)	148 (64 - 150)	0,282 ^m^
FEVE (Mediana, FIQ1- FIQ3)			61 (60 - 66)	60 (62,8 - 66)	0,155 ^m^
AE, (mm) (Mediana, FIQ1- FIQ3)			36 (35 - 37)	43 (38 - 47)	<0,001 ^m^
PSAP (mmHg) (Mediana, FIQ1- FIQ3)			20 (18 - 20)	31 (27,8 – 39,3)	<0,001 ^m^
RM Mo/G		Ausente	54 (100%)	60 (65%)	
		Presente	0 (0%)	32 (35%)	
EMi Grave		Ausente	54 (100%)	77 (84%)	
		Presente	0 (0%)	15 (16%)	
Histórico de VMPB			0 (0%)	15 (16%)	
TV cirúrgica			0 (0%)	0 (0%)	
AVM			-	2,2 ± 1,3	
DVAR			0 (0%)	47 (51%)	

*IMC: Índice de massa corporal; DM: diabetes mellitus; HDL: lipoproteína de alta densidade; HT: hipertensão; AE: átrio esquerdo; LDL: lipoproteína de baixa densidade; FEVE: fração de ejeção ventricular esquerda; DVMR: doença valvar mitral reumatismal; PSAP: pressão sistólica da artéria pulmonar; WBC: leucócitos; RM Mo/G: Regurgitação mitral moderada ou grave; VMPB: valvoplastia mitral percutânea por balão; TV: troca valvar; AVM: área valvar mitral; DVAR: doença valvar aórtica reumática (estenose ou regurgitação). ^m^ Teste U de Mann-Whitney, ^X^2^^ Teste Qui-quadrado (χ2).*

Os níveis de tiol nativo (407±83 μmol/L vs. 297±65 μmol/L, p<0,001) e tiol total (442±82 μmol/L vs. 329±65 μmol/L, p<0,001) são mais altos no grupo de controle. Os níveis de dissulfeto (16,7±4,9 μmol/L vs. 14,8±3,7 μmol/L, p=0,011) são elevados no grupo de pacientes com DVMR. As razões dissulfeto/tiol total e as razões dissulfeto/tiol nativo médias são mais altas no grupo de pacientes com DVMR, enquanto as razões tiol nativo/dissulfeto e as razões tiol total/dissulfeto são mais altas no grupo de controle. Os níveis de tiol nativo, tiol total, dissulfeto, razão dissulfeto/tióis e razão tióis/dissulfeto entre pacientes com ou sem DVMR são apresentadas na Tabela 2 .

**Tabela 2:** – Nível de tiol nativo, tiol total, dissulfeto, razão dissulfeto/tióis e razão tióis/dissulfeto entre pacientes com ou sem doença valvar mitral reumatismal

	Controle (n:54)	DVMR (n:92)	p
Tiol total, (mmol/L) (Média ± DP)	442±82	329±65	<0,001 ^t^
Tiol nativo, (mmol/L) (Média ± DP)	407±83	298± 65	<0,001 ^t^
Dissulfeto, (mmol/L) (Mediana, FIQ1- FIQ3)	15,1 (13,4 – 17,6)	17 (14,8 - 19,9)	0,011 ^m^
Dissulfeto/tiol total, %x100 (Mediana, FIQ1- FIQ3)	3,4 (2,8 - 4)	5,4 (4,3 - 6,6)	<0,001 ^m^
Dissulfeto/tiol nativo, %x100 (Mediana, FIQ1- FIQ3)	3,7 (3 - 4,4)	5,8 (4,7 – 7,4)	<0,001 ^m^

*DVMR: doença valvar mitral reumatismal; ^m^ Teste U de Mann-Whitney; ^t^ Teste t de Student.*

A análise de correlação mostrou que houve correlação positiva entre os níveis de dissulfeto e a gravidade da estenose mitral (área da válvula mitral <1,5 cm^2^); entre a razão dissulfeto/tiol total e nativo com PSAP, diâmetro de AE, e gravidade da estenose mitral. Além disso, houve correlações negativas entre o tiol nativo e o tiol total com PSAP, diâmetro de AE, e gravidade da estenose mitral. A análise de correlação entre os parâmetros de tiol e dissulfeto com achados de ecocardiogramas está listada na Tabela 3 .

**Tabela 3:** – Análise da correlação entre parâmetros de tiol e de dissulfeto com achados ecocardiográficos

		PSAP	EMi Grave	AE
Tiol nativo	Rô de Spearman	-0,598	-0,319	-0,532
	p	<0,001	<0,001	<0,001
Tiol total	Rô de Spearman	-0,596	-0,300	-0,549
	p	<0,001	<0,001	<0,001
Dissulfeto	Rô de Spearman	0,135	0,188	0,107
	p	0,106	0,023	0,201
Dissulfeto/tiol nativo	Rô de Spearman	0,469	0,331	0,403
	p	<0,001	<0,001	<0,001
Dissulfeto/tiol total	Rô de Spearman	0,473	0,333	0,405
	p	<0,001	<0,001	<0,001

*AE: átrio esquerdo; EMi: estenose da válvula mitral; PSAP: pressão sistólica da artéria pulmonar.*

O nível de tiol nativo, tiol total, dissulfeto, razão dissulfeto/tióis e razão tióis/dissulfeto entre pacientes com ou sem estenose mitral reumática grave (EMRG) está listado na Tabela 4 . Não houve uma diferença significativa em tiol nativo, tiol total, e dissulfeto entre os pacientes que tinham EMi leve a moderada e EMi grave ou histórico de valvoplastia mitral percutânea. Entretanto, as razões de dissulfeto/tiol total e dissulfeto/tiol nativo foram significativamente mais altas em pacientes com EMi grave ou histórico de valvoplastia mitral percutânea. Por outro lado, não foram observadas diferenças significativas nos níveis de tiol nativo, tiol total, dissulfeto, e nas razões dissulfeto/tiol total e dissulfeto/tiol nativo de acordo com a gravidade de regurgitação mitral do paciente.

**Tabela 4:** – Nível de tiol nativo, tiol total, dissulfeto, razão dissulfeto/tióis e razão tióis/dissulfeto entre pacientes com ou sem estenose mitral reumática

	EMi leve - moderada (n:62)	EMi grave ou Histórico de VMPB (n:30)	p
Tiol total, (mmol/L) (Média ± DP)	335±66	317±62	0,125 ^t^
Tiol nativo, (mmol/L) (Média ± DP)	304±67	282±59	0,215 ^t^
Dissulfeto, (mmol/L) (Média ± DP)	17±5	19±7	0,093 ^t^
Dissulfeto/tiol total, %x100 (Média ± DP)	5,2±1,7	6,1±2,1	0,045 ^t^
Dissulfeto/tiol nativo, %x100 (Média ± DP)	5,8±2,1	7,1±3,7	0,048 ^t^

*EMi: Estenose mitral; VMPB: valvoplastia mitral percutânea por balão; ^m^ Teste U de Mann-Whitney; ^t^ Teste t de Student.*

## Discussão

Os níveis de tiol plasmático foram significativamente mais baixos em pacientes com DVMR em comparação com o grupo de controle. Os níveis de dissulfeto e a razão dissulfeto/tióis foram maiores em pacientes com DVMR. Até onde se sabe, este é o único estudo que avaliou a homeostase tiol/dissulfeto como um novo preditor, que está relacionado de forma mais próxima à DVMR e à gravidade da EMi.

A doença cardíaca reumática e a DVMR são as complicações graves da febre reumática aguda e causa lesões valvares crônicas, levando a morbidade e mortalidade.^2^ A DCR é responsável por uma parte crucial da carga de saúde em muitos países em desenvolvimento.^19^ A DVMR tem um mecanismo complexo em que o principal é a inflamação crônica e as reações autoimunes.

Estudos anteriores apresentaram evidências convincentes de que houve uma inflamação progressiva na DCR, e essa inflamação persistente já causou danos ao tecido valvar.^7^ Vários estudos avaliaram marcadores de inflamação crônica em pacientes com DCR. Alguns desses estudos demonstram que níveis séricos mais elevados de PCR ultrassensível e Pentraxina-3, em comparação com sujeitos saudáveis, poderiam ser considerados marcadores de inflamação em pacientes com DVMR.^20 , 21^ Em uma pesquisa recente, a razão neutrófilo-linfócito (NLR) era significativamente mais alta em pacientes com DVMR grave que em pacientes com DVMR leve a moderada.^22^

Estudos recentes demonstraram a possibilidade de interações entre as interleucinas e a inflamação crônica, inclusive com o desenvolvimento de DCR. Davutoglu et al. e Bilik et al. demonstraram que pacientes com DVMR tinham níveis plasmáticos mais elevados de fator de necrose tumoral alfa (TNFa), IL-2, IL-6, IL-8, e IL-17, IL-23 como preditores de inflamação progressiva e resposta autoimune que sujeitos saudáveis.^23 , 24^ Em estudos anteriores, marcadores de stress oxidativo no tecido e no plasma foram investigados em pacientes com DCR. Ele determinou que os níveis de produtos proteicos de oxidação avançada eram mais altos em pacientes com DCR que no grupo de controle.^25 , 26^ Além disso, nosso estudo foi o primeiro a avaliar o papel da razão tiol-dissulfeto e marcadores de stress oxidativo inéditos em pacientes com DCR.

O stress oxidativo (SO) é o desequilíbrio entre espécies reativas do oxigênio (ERO) e substâncias antioxidantes, e ele pode ser tóxico às células levando à peroxidação lipídica da membrana e lesão à membrana.^27 , 28^ Os tióis são antioxidantes significativos e desempenham um papel importante na eliminação não enzimática de ERO.^10 , 13^ Embora as oxidações de tióis proteicos tenha sido considerada uma reação colateral indesejável do stress oxidativo, a identificação de proteínas redox reguladas mostrou que modificações reversíveis do tiol foram importantes para ajustar sua atividade às condições de redox prevalentes no ambiente.^29^ A definição da importância funcional de modificações de tiol continua a ser um desafio significativo no campo e ainda exige que estudos bioquímicos sejam feitos em casos e doenças diferentes.

Recentemente, a significância da homeostase dissulfeto/tiol foi demonstrada em vários estudos. Um destes, realizado por Kundi et al.,^14^ demonstrou que a razão dissulfeto/tiol aumentava no IAM, e os autores concluíram que esse valor poderia ser usado como preditor para a detecção de lesão miocárdica aguda.^14^ Em outro estudo, Topuz et al. demonstraram que a homeostase tiol/dissulfeto pode se alterar durante o tromboembolismo pulmonar agudo. Além disso, ela pode estar relacionada a medições hemodinâmicas comprometidas.^30^ Vários estudos relatam que a redução das concentrações de tiol e da razão tiol/dissulfeto pode ser um fator crucial no desenvolvimento da aterosclerose, da ectasia da artéria coronária (EAC), e da toxicidade cardíaca induzida por quimioterapia.^15 , 31 , 32^ Resumidamente, os autores concluíram que o stress oxidativo pode envolver o fator principal de patogênese. Em nosso estudo, os níveis de tiol e a razão tiol/dissulfeto estão relacionados à DVMR e à gravidade do envolvimento mitral reumático, confirmando a hipótese de que a válvula reumática seja a causa do stress oxidativo além da inflamação crônica.

Até onde se sabe, este é o primeiro estudo a avaliar a relação entre a homeostase tiol/dissulfeto e seus impactos na DCR, e a intensidade do dano à válvula em pacientes com DVMR. Nossos achados mostram que a homeostase tiol/dissulfeto poderia ter um papel essencial na fisiopatologia da lesão à válvula reumática. Informações definitivas sobre o assunto poderiam ser conseguidas por meio de estudos de tecido in vitro e in vivo.

### Limitações

Este estudo tem várias limitações. Primeiramente, uma amostra relativamente pequena restringiu a possibilidade de generalização dos achados de nossa pesquisa. A falta de dados de monitoramento e alterações em série de tióis, e a ausência de medições simultâneas de outros mediadores inflamatórios e autoimunes inéditos são outras limitações do estudo. Os valores de tiol/dissulfeto não são comparáveis a outros marcadores enzimáticos e não enzimáticos de stress oxidativo. Finalmente, o estudo poderia ser melhorado, acrescentando-se o polimorfismo genético a pacientes com DVMR.

## Conclusões

O presente estudo é o primeiro a demonstrar a associação entre os níveis de tiol e a homeostase de tiol/dissulfeto em pacientes com DVMR. Nossos achados demonstraram seu possível papel na gravidade do dano à válvula na fisiopatologia da DVMR.
