# Supplementary material for: Níveis de Tiol Sérico e Homeostase Tiol/Dissulfeto em Pacientes com Doença Valvar Mitral Reumatismal e em Sujeitos Saudáveis
Source: Arq Bras Cardiol. 2021 Jul 27;117(3):437–43. [Article in Portuguese] doi: 10.36660/abc.20200161 (PMC8462953; doi:10.36660/abc.20200161)
Supplement: Translation

# Translation: Serum Thiol Levels and Thiol/Disulfide Homeostasis in Patients with Rheumatic Mitral Valve Disease and Healthy Subjects

**Authors:** Ahmet Korkmaz, Birsen Doğanay, Funda Basyigit, Cem Çöteli, Abdulkadir Yildiz, Tugba Gursoy, Umit Guray, Ozgul Ucar Elalmis

**Affiliations:** 1 Turkey Ministry of Health Ankara City Hospital - Department of Cardiology, Ankara - Turkey; 2 Turkey Ozel Anadolu Hastanesi - Cardiology Clinic, Kastamonu - Turkey

**Keywords:** Rheumatic Diseases, Mitral Valve Stenosis, Homeostasis, Tiol/Dissulfide, Echocardiography/methods, Oxidative Stress

## Abstract

**Background:**

Rheumatic mitral valve disease (RMVD) is the most common presentation of rheumatic heart disease (RHD). Inflammation and fibrosis processes also play significant roles in its pathogenesis. Recent studies showed that thiols and thiol-disulfide are promising novel oxidative stress markers.

**Objectives:**

The present study aimed to evaluate differences in the serum thiol and thiol-disulfide levels in patients with RMVD and the control group.

**Methods:**

Ninety-two patients with RMVD were enrolled in the study. Fifty-four healthy subjects, age, and gender-matched with the study group, were also included in the study as a control group. This study investigated thiol levels in patients with RMVD and the control group. P-values lower than 0.05 were considered statistically significant.

**Results:**

The patients with RMVD presented higher systolic pulmonary artery pressure (SPAP) and left atrial (LA) diameter levels than the control group. Native thiol (407±83 μmol/L vs. 297±65 μmol/L, p<0.001) and total thiol (442±82 μmol/L vs. 329±65 μmol/L, p<0.001) levels were higher in the control group. Disulfide (16.7±4.9 μmol/L vs. 14.8±3.7 μmol/L, p=0.011) levels were higher in the group of patients with RMVD. A positive correlation was found between disulfide/native and disulfide/total thiols ratio with SPAP, LA diameter, and MS severity. Disulfide/total thiols ratio was significantly higher in patients with severe MS than with mild to moderate MS patients.

**Conclusions:**

To the best of our knowledge, this is the only study of its kind that has evaluated thiol/disulfide homeostasis as a novel predictor, which was more closely related to RMVD and the severity of MS.

## Introduction

Rheumatic heart disease (RHD) is a widely observed cardiovascular disease in children and adolescents.^1^ Rheumatic mitral valve disease (RMVD) is the most common presentation of RHD.^2^ A study in 2015 reported that the estimated number of RHD patients was 33.4 million worldwide.^3^ In the PROVAR study, the authors screened 5,996 students, whose median age was 11.9 (range 9.0 to 15.0), with echocardiography. The authors reported that RHD prevalence was 42/1,000 in Brazilian children.^4^

Rheumatic heart disease occurs after an autoimmune reaction, which is triggered by an untreated streptococcal upper respiratory tract infection. This process causes severe valvular injury in genetically susceptible subjects.^5^ Despite the unknown pathophysiology of RHD, there are hypotheses that several autoimmune and inflammatory reactions, oxidative stress, immune system genes, and polymorphisms are related to RHD.^5 - 9^

Thiols are crucial antioxidant agents in human physiology. Thiol concentrations are lower in plasma. This occurs because thiols mostly consist of human plasma albumin with low-molecular-weight thiols, including cysteine (Cys), homocysteine, glutathione, cysteinyl glycine, and γ-glutamyl-cysteine.^10^ The oxidative processes can transform thiols into many different molecules. The thiol-disulfide is one of the products of the oxidative reactions in which thiols are involved.^11^ The oxidation of Cys residues may produce a reversible production of various disulfides, such as low-molecular-mass thiols and protein thiol molecules. Furthermore, disulfide residues can be converted into thiol groups to keep the thiol/disulfide homeostasis stable.^12^ Therefore, thiols consist of a crucial part of the total amount of antioxidants and play a significant role in the antioxidant mechanism for radical oxygen species (ROS).^13^

The current studies demonstrated that the thiol-disulfide ratio had significant value as a promising oxidative stress marker.^14 , 15^ Although the importance of thiol metabolism was evaluated in different cardiovascular diseases and procedures, such as myocardial infarction or coronary artery bypass surgery, its expression in RHD is unknown.^14 , 16^ This study, due to the pathophysiological component of RVD, aimed to evaluate the thiol levels in RMVD patients and healthy subjects.

## Methods

Ninety-two patients with RMVD who were admitted to our cardiology clinic between April 2018 and December 2019 were enrolled in the study. Fifty-four healthy subjects, matched to patients, were also included in the study as a control group. Gender, age, body mass index (BMI), comorbidities, left ventricle ejection fraction, and smoking status were considered for the pairing of the groups.

Written informed consent was obtained for all participants. The exclusion criteria were patients with Marfan syndrome, bicuspid aortic valve, non-rheumatic mitral valve pathologies, or prior open-heart surgery. Furthermore, patients with liver, thyroid, and kidney diseases, blood disorders, connective tissue or inflammatory disease, any history of cancer, and acute or chronic infection were also excluded from the study.

The relevant demographic, anthropometric, and medical history data were recorded. Clinical information, such as Framingham’s coronary risk factors (hypertension (HT), diabetes mellitus (DM), smoking, hyperlipidemia, and family history of coronary disease history), were also collected.

Transthoracic two-dimensional and color flow Doppler echocardiography was used in all patients using ultrasound with 2.5-MHz transducers (Toshiba SSH160A). The M-mode echocardiography was used to measure the left atrial diameter, and the planimetric and pressure half time methods to assess the mitral valve area. The transmitral gradient was defined with a continuous wave Doppler in an apical four-chamber view. Colour-flow Doppler was performed to observe the presence and severity of mitral regurgitation (MR). Pulmonary artery systolic pressure was measured by continuous-wave Doppler studies using the Bernoulli equation. The criteria for the diagnosis of RMVD included the mitral valve area ≤ 2.5 cm^2^ , the presence of leaflet thickening, commissural fusion, and changing in the subvalvular area, detected by an echocardiogram.^17^

Blood samples were drawn from the antecubital vein during hospital admission. The blood samples of the patients and control groups were taken in the morning after a 12-hour fasting period. All blood samples were collected into tubes containing no additives. Serum was obtained after centrifugation at 1,500 g for 10 minutes and stored at -80^0^C until analysis.

Thiol/disulfide homeostasis was conducted by the procedure determined by Erel et al.^18^ Subsequently, free functional thiol groups were obtained with the reduction of disulfide bonds. Sodium borohydride was applied as a reductant, and unused reductant was removed by formaldehyde. After 5, 5′-dithiobis-(2-nitrobenzoic) acid reaction, all thiol groups, both native and reduced, were defined. Dynamic disulfide quantity (−S-S) was confirmed by half of the difference between the native and total thiols. After evaluating the magnitude of native thiol (−SH) and disulfide (−S-S), the disulfide/native thiol (−S-S−/−SH) ratio was calculated.^18^

### Statistical Analysis

The normality of data was analyzed using the Kolmogorov-Smirnov test. Quantitative variables with normal distribution were presented as mean and standard deviation. The variables with non-normal distribution were presented as median (interquartile range), and categorical data as number and percentage. Independent samples t-test was used to compare groups for continuous data with normal distribution, whereas the Mann-Whitney U test was performed for variables with non-normal distribution. Categorical data were analyzed using the Chi-square or Fisher’s Exact tests. The relations among the numerical and categorical variables were analyzed with Spearman correlation analysis. Differences were accepted as significant at the two-sided p<0.05 level. All statistical analysis was carried out using the Statistical Package for Social Sciences program (SPSS) for Windows version 22 (IBM SPSS Inc., Chicago, IL).

## Results

A total of 146 subjects, of which 92 had RMVD and 54 did not, were included in the study. The demographic, clinical, and laboratory data of the study groups are displayed in Table 1 . In patients with RMVD, a total of 22 (24%) were male and 70 (76%) were female, whereas 15 (28%) patients were male and 39 (72%) were female in the control group. The mean age was 48±10 years and 46.7±11.2 years in the patients with RMVD and control groups, respectively. No differences were found between the groups in terms of age, gender, BMI, HT, DM, smoking, and other laboratory parameters. As expected, the patients with RMVD had higher systolic pulmonary artery pressure (SPAP) and left atrial (LA) diameter levels than in the control group.

**Table 1:** – Baseline Clinical, Demographic, and Laboratory Characteristics of The Patients with and without Rheumatic Mitral Valve Disease

			Control (n:54)	RMVD (n:92)	p
Age (Median,IQR1-IQR3)			45 (39.3 - 54.5)	48 (42 - 55.8)	0.304^m^
Sex (n,%)	Male		15 (28%)	22 (24%)	0.604^X²^
	Female		39 (72%)	70 (76%)	
DM (n,%)	No		48 (89%)	88 (96%)	0.133^X²^
	Yes		6 (11%)	4 (4%)	
HT (n,%)	No		46 (85%)	79 (86%)	0.492^X²^
	Yes		8 (15%)	13 (14%)	
Smoking (n,%)	No		40 (74%)	58 (63%)	0.134^X²^
	Yes		14 (26%)	34 (37%)	
BMI (Median,IQR1-IQR3)			24 (22 - 25.8)	24 (21.9 - 25)	0.139^m^
Glucose (mg/dL) (Median,IQR1-IQR3)			94 (88.5 - 102)	90 (21.9 - 25)	0.054^m^
Serum creatinine (mg/dL) (Median,IQR1-IQR3)			0.8 (0.6 - 0.8)	0.7 (0.6 - 0.8)	0.128^m^
Hemoglobin(g/L) (Median,IQR1-IQR3)			14 (13 - 14)	13.8 (12 - 14)	0.509^m^
WBC count (x1000/mm3) (Median,IQR1-IQR3)			6.4 (5.9 - 7.3)	6.2 (6 - 7)	0.423^m^
Platelet count (x1000/mm3) (Median,IQR1-IQR3)			300 (254.5 - 348.8)	301 (277 - 313)	0.766^m^
Total cholesterol (mg/dL) (Median,IQR1-IQR3)			215 (195.3 - 235)	197 (195 - 225)	0.075^m^
LDL, (mg/dL) (Median,IQR1-IQR3)			120 (103.3 - 125)	114 (100 - 121)	0.086^m^
HDL, (mg/dL) (Median,IQR1-IQR3)			49 (45 - 55.8)	51 (45.8 - 72)	0.113^m^
Triglycerides (mg/dL) (Median,IQR1-IQR3)			150 (90.8 - 185)	148 (64 - 150)	0.282^m^
LVEF,(%) (Median,IQR1-IQR3)			61 (60 - 66)	60 (62.8 - 66)	0.155^m^
LA, (mm) (Median,IQR1-IQR3)			36 (35 - 37)	43 (38 - 47)	<0.001^m^
SPAP, (mmHg) (Median,IQR1-IQR3)			20 (18 - 20)	31 (27.8 – 39.3)	<0.001^m^
Mo/S MR		Absent	54 (100%)	60 (65%)	
		Present	0 (0%)	32 (35%)	
Severe MS		Absent	54 (100%)	77 (84%)	
		Present	0 (0%)	15 (16%)	
P-MBVP History			0 (0%)	15 (16%)	
Surgical VR			0 (0%)	0 (0%)	
MVA			-	2.2 ± 1.3	
RAVD			0 (0%)	47 (51%)	

*BMI: body mass index; DM: diabetes mellitus; HDL: high-density lipoprotein; HT: hypertension; LA: left atrium; LDL: low-density lipoprotein; LVEF: left ventricular ejection fraction; RMVD: rheumatic mitral valve disease; SPAB: systolic pulmonary artery pressure; WBC: white blood cell; Mo/S MR: moderate or severe mitral regurgitation; P-MBVP: prcutaneous mitral balloon valvuloplasty; VR: valve replacement; MVA: mitral valve area; RAVD: rheumatic aortic valve disease (Stenosis or Regurgitation).^m^Mann-Whitney U Test,^X^2^^Chi Square (χ2) Test*

Native thiol (407±83 μmol/L vs. 297±65 μmol/L, p<0.001) and total thiol (442±82 μmol/L vs. 329±65 μmol/L, p<0.001) levels were higher in the control group. Disulfide (16.7±4.9 μmol/L vs. 14.8±3.7 μmol/L, p=0.011) levels were elevated in patients with RMVD group. The mean disulfide/total thiol ratios and disulfide/native thiol ratios were higher in patients with RMVD group, while native thiol/disulfide ratios, and total thiol/disulfide ratios were higher in the control group. The levels of native thiol, total thiol, disulfide, disulfide/thiols, and thiols/disulfide ratio between the patients with and without RMVD are shown in Table 2 .

**Table 2:** – The Level of Native Thiol, Total Thiol, Disulfide, Disulfide/Thiols, and Thiols/Disulfide Ratio Between the Patients with and without Rheumatic Mitral Valve Disease

	Control (n:54)	RMVD (n:92)	p
Total Thiol, (mmol/L) (Mean ± SD)	442±82	329±65	<0.001^t^
Native Thiol, (mmol/L) (Mean ± SD)	407±83	298± 65	<0.001^t^
Disulfide, (mmol/L) (Median,IQR1-IQR3)	15.1 (13.4 - 17.6)	17 (14.8 - 19.9)	0.011^m^
Disulfide/Total Thiol, %x100 (Median,IQR1-IQR3)	3.4 (2.8 - 4)	5.4 (4.3 - 6.6)	<0.001^m^
Disulfide/Native Thiol, %x100 (Median,IQR1-IQR3)	3.7 (3 - 4.4)	5.8 (4.7 - 7.4)	<0.001^m^

*RMVD: Rheumatic mitral valve disease;^m^Mann-Whitney U Test;^t^t -test*

Correlation analysis showed that there were positive correlations between disulfide levels and mitral stenosis severity (mitral valve area <1.5 cm^2^ ); between the disulfide/total and the native/thiol ratio with SPAP, LA diameter, and mitral stenosis severity. Furthermore, there were negative correlations between native thiol and total thiol with SPAP, LA diameter, and mitral stenosis severity. Correlation analysis of thiol and disulfide parameters with echocardiographic findings is listed in Table 3 .

**Table 3:** – Correlation Analysis of Thiol and Disulfide Parameters with Echocardiographic Findings

		SPAB	Severe MS	LA
Native thiol	Spearman’s Rho	-.598	-.319	-.532
	P	<0.001	<0.001	<0.001
Total thiol	Spearman’s Rho	-.596	-.300	-.549
	P	<0.001	<0.001	<0.001
Disulfide	Spearman’s Rho	.135	.188	.107
	P	0.106	0.023	0.201
Disulfide / Native thiol	Spearman’s Rho	.469	.331	.403
	P	<0.001	<0.001	<0.001
Disulfide / Total thiol	Spearman’s Rho	.473	.333	.405
	P	<0.001	<0.001	<0.001

*LA: Left atrium; MS: mitral valve stenosis; SPAB: Systolic pulmonary artery pressure*

The level of native thiol, total thiol, disulfide, disulfide/thiols, and thiols/disulfide ratio between the patients with and without severe rheumatic mitral valve stenosis (RMVS) is listed in Table 4 . No significant difference was observed in native thiol, total thiol, and disulfide between the patients who had mild to moderate MS and severe MS or percutaneous mitral valvuloplasty history. However, disulfide/total and disulfide/native thiols ratios were significantly higher in patients with severe MS or percutaneous mitral valvuloplasty history. On the other hand, any significant difference in native thiol, total thiol, disulfide levels, and disulfide/total and disulfide/native thiols ratios was not observed according to the patients’ severity of mitral regurgitation.

**Table 4:** – The Level of Native Thiol, Total Thiol, Disulfide, Disulfide/Thiols and Thiols/Disulfide Ratio Between the Patients with and without Severe Rheumatic Mitral Valve Stenosis

	Mild-Moderate MS (n:62)	Severe MS or P-MBVP History (n:30)	p
Total Thiol, (mmol/L) (Mean ± SD)	335±66	317±62	0.125^t^
Native Thiol, (mmol/L) (Mean ± SD)	304±67	282±59	0.215^t^
Disulfide, (mmol/L) (Mean ± SD)	17±5	19±7	0.093^t^
Disulfide/Total Thiol, %x100 (Mean ± SD)	5.2±1.7	6.1±2.1	0.045^t^
Disulfide/Native Thiol, %x100 (Mean ± SD)	5.8±2.1	7.1±3.7	0.048^t^

*MS: Mitral Stenosis; P-MBVP: Percutaneous Mitral Balloon Valvuloplasty;^m^Mann-Whitney U Test;^t^t -test*

## Discussion

Plasma thiol levels were significantly lower in patients with RMVD, when compared to the control group. Disulfide levels and the disulfide/thiols ratio were higher in patients with RMVD. To th best of our knowledge, this is the only study that has evaluated thiol/disulfide homeostasis as a new predictor, which was more closely related to RMVD and the severity of MS.

RHD and RMVD are the severe complications of acute rheumatic fever and lead to chronic valvular lesions causing the morbidity and mortality.^2^ RHD holds a crucial part of the health burden in many developing countries.^19^ RMVD has a complex mechanism, the main ones of which are chronic inflammation and autoimmune reactions.

The previous studies have reported convincing evidence that there has been a progressive inflammation in RHD, and this persistent inflammation has led to damage to the valvular tissue.^7^ Several studies assessed chronic inflammation markers in RHD patients. Some of these studies showed that elevated serum levels of hsCRP and Pentraxin-3 could be considered as markers of inflammation in RMVD patients than in healthy subjects.^20 , 21^ In a recent survey, the neutrophil-to-lymphocyte ratio (NLR) was significantly higher in patients with severe RMVD than in patients with mild to moderate RMVD.^22^

Recent studies have shown that there were possible interactions between interleukins and chronic inflammation, including RHD development. Davutoglu et al. and Bilik et al. demonstrated that patients with RMVD had elevated plasma levels of tumor necrosis factor-alpha (TNFa), IL-2, IL-6, IL-8, and IL-17, IL-23 as predictors of progressive inflammation and autoimmune response than in healthy subjects.^23 , 24^ In previous studies, plasma and tissue oxidative stress markers were investigated in patients with RHD. They determined that levels of advanced oxidation protein products were higher in RHD patients than in controls.^25 , 26^ Furthermore, our study was the first to assess novel oxidative stress markers thiol-disulfide ratio role in patients with RHD.

Oxidative stress (OS) is the imbalance between reactive oxygen species (ROS) and antioxidant substances; it may be toxic to cells by leading to membrane lipid peroxidation and membrane injury.^27 , 28^ Thiols are significant antioxidants and play a substantial role in the non-enzymatic elimination of ROS.^10 , 13^ Although protein thiol oxidations have been considered undesirable side reactions of oxidative stress, the identification of redox-regulated proteins showed that reversible thiol modifications were important to adjust their activity to the prevailing redox conditions of the environment.^29^ Defining the functional importance of thiol modifications remains a significant challenge in the field, and still requires biochemical studies in different cases and diseases.

Recently, the significance of disulfide/thiol homeostasis has been demonstrated by various studies. One of these studies, by Kundi et al.,^14^ showed that the disulfide/thiol ratio elevated in AMI, and the authors concluded that this value could be used as a predictor for the detection of acute myocardial injury.^14^ In another study, Topuz et al. demonstrated that thiol/disulfide homeostasis might change during acute pulmonary thromboembolism. Moreover, this might be related to impaired hemodynamic measurements.^30^ Several studies reported that decreased thiol concentrations and thiol/disulfide ratio might be a crucial factor in the development of atherosclerosis, coronary artery ectasia (CAE), and chemotherapy-induced cardiac toxicity.^15 , 31 , 32^ Briefly, the authors concluded that oxidative stress may well involve the main factor of pathogenesis. In our study, thiol levels and the thiol/disulfide ratio are related to RMVD and the severity of rheumatic mitral valve involvement, confirming the hypothesis that the rheumatic valve is the cause of oxidative stress in addition to chronic inflammation.

To the best of our knowledge, this is the first study to evaluate the relationship between thiol/disulfide homeostasis and its impacts on RHD, as well as the intensity of valve damage in patients with RMVD. Our findings showed that thiol/disulfide homeostasis could play an essential role in the pathophysiology of rheumatic valve damage. Definitive information about the topic could be provided with in-vivo and in-vitro tissue studies.

### Limitations

The present study has several limitations. First, a relatively small sample size restricted the generalizability of the findings of our research. The lack of follow-up data and serial changes in thiols, and the absence of simultaneous measurement of another novel autoimmune and inflammatory mediators are other limitations. Thiol/disulfide values did not compare with other enzymatic and non-enzymatic oxidative stress markers. Finally, the study could be enhanced by adding genetic polymorphisms in patients with RMVD.

## Conclusions

The present study is the first to show the association between thiol levels and thiol/disulfide homeostasis in patients with RMVD. Our findings demonstrated its possible role in the severity of valve damage and in the pathophysiology of RMVD.
